# Epidemic spreading and control strategies in spatial modular network

**DOI:** 10.1007/s41109-020-00337-4

**Published:** 2020-11-26

**Authors:** Bnaya Gross, Shlomo Havlin

**Affiliations:** Department of Physics, Bar-Ilan University, 52900 Ramat-Gan, Israel

**Keywords:** Epidemic spreading, Control strategies, Spatial networks, Community networks, Modular networks

## Abstract

Epidemic spread on networks is one of the most studied dynamics in network science and has important implications in real epidemic scenarios. Nonetheless, the dynamics of real epidemics and how it is affected by the underline structure of the infection channels are still not fully understood. Here we apply the susceptible-infected-recovered model and study analytically and numerically the epidemic spread on a recently developed spatial modular model imitating the structure of cities in a country. The model assumes that inside a city the infection channels connect many different locations, while the infection channels between cities are less and usually directly connect only a few nearest neighbor cities in a two-dimensional plane. We find that the model experience two epidemic transitions. The first lower threshold represents a local epidemic spread within a city but not to the entire country and the second higher threshold represents a global epidemic in the entire country. Based on our analytical solution we proposed several control strategies and how to optimize them. We also show that while control strategies can successfully control the disease, early actions are essentials to prevent the disease global spread.

## Introduction

Network science is becoming one of the most fruitful research fields in the last decades explaining variety of phenomena in many complex systems such as the human brain (Moretti and Muñoz 2013; Sporns 2010) the human microbiome (Smillie et al. 2011; Gibson et al. 2016; Layeghifard et al. 2017), protein-protein interactions (Kovács et al. 2019; De Domenico et al. 2015; Li et al. 2017), climate (Yamasaki et al. 2008; Fan et al. 2017; Ludescher et al. 2014), ecology (Paine 1966; Polis and Strong 1996) and infrastructures (Yang et al. 2017; Latora and Marchiori 2005; Li et al. 2015). Modelling of these systems and many others opened a new direction of studying many complex structures such as modular (community) networks (Palla et al. 2005; Rosvall and Bergstrom 2008; Gross et al. 2020b; Capocci et al. 2005; Shekhtman et al. 2015; Girvan and Newman 2002), multiplex networks (Nicosia et al. 2013; Gomez et al. 2013; Granell et al. 2013; Bianconi 2013), interdependent networks (Wang et al. 2013; Buldyrev et al. 2010; Brummitt et al. 2012; Baxter et al. 2012; Gao et al. 2012; Radicchi and Arenas 2013) and high order interactions networks (Lambiotte et al. 2019; de Arruda et al. 2020; Millán et al. 2020). These structures were studied under different processes and dynamics such as percolation (Bunde and Havlin 1991; Stauffer and Aharony 2018), synchronization (Arenas et al. 2006; Danziger et al. 2019; De Domenico 2017), reaction-diffusion (Weber et al. 2008; Cencetti et al. 2018; Lazaridis et al. 2018; Colizza et al. 2007), and epidemics (Pastor-Satorras et al. 2015; Boguá et al. 2003; Wang et al. 2017).

When modeling a process, one should be careful not strictly fitting the model to a specific scenario which will reduce its generality, and to account for as many as possible of the important features of the process in order to make the model valid and useful in different scenarios. While recently the study of epidemic spread has been conducted on a community structure due to the human social organization (Palla et al. 2007; Jin et al. 2001), it mainly considered a random organization of the communities while neglecting the spatial structure (Salathé and Jones 2010; Valdez et al. 2020; Nadini et al. 2018; Liu and Hu 2005).

**Fig. 1:**
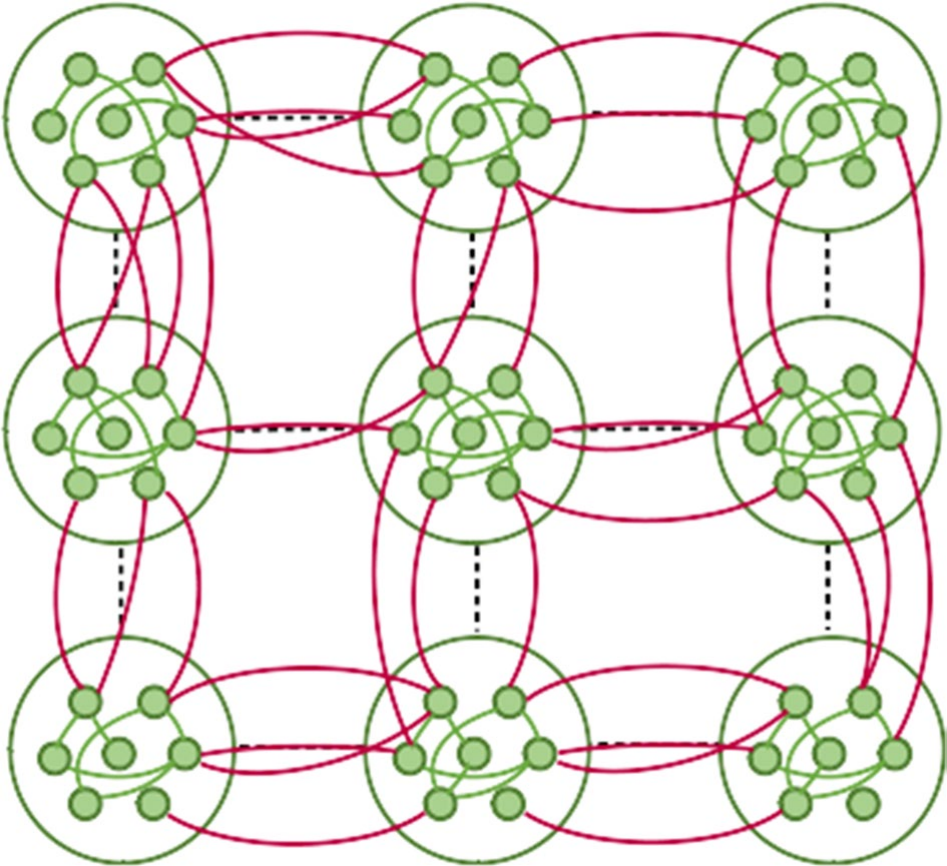
Illustration of the model. The spatial modular model represents a structure of a network of infection channels inside cities (modules) and between cities. Inside a city, the infection channels are dense and spread randomly between different areas of the city (green links) like in an Erdős–Rényi network having random like structure while the infection channels from one city to another is usually possible between neighbouring cities (red links) having spatial like structure

In this paper, we applied the susceptible-infected-recovered (SIR) model to study the epidemic spreading in a 2D spatial community network model (Vaknin et al. 2019; Gross et al. 2020c), see Fig. 1, to better describe epidemic spreading in human social community organization. Each community can represent a city and the entire network represents a country. While other epidemic models such as agent-based models (Eubank et al. 2004; Longini et al. 2005; Ferguson et al. 2005, 2006) and metapopulation models (Ajelli et al. 2010; Colizza and Vespignani 2008; Juher et al. 2009; Rvachev and Longini 1985; Colizza et al. 2006; Balcan et al. 2009) are widely used and allow tracking of each individual trajectory, we show here that the basic SIR model show a rich phenomena of how the spatial modular structure affects the epidemic spreading.

We find that the epidemic spreading in such networks experience two epidemic transitions, a phenomena which not been earlier observed in other spatial dynamic and epidemic models (Fernández-Gracia et al. 2014; Braha 2012; Smith et al. 2002; Durrett 1995). The first transition is observed at $\beta ^{ER}_c$ when a local outbreak spread in the origin city but not in the entire country, and the second at $\beta ^{2D}_c$ when the epidemic spreads in the entire country. We find analytically the values of both epidemic thresholds and develop several control strategies and optimization methods to mitigate the spreading of the disease. Moreover, we show the importance of early actions and how delaying might result in a global spread of the epidemic with catastrophic results.

## Model

The spatial community model (Vaknin et al. 2019; Gross et al. 2020c) illustrated in Fig. 1, represents the infection channels within and between communities on a 2-dimensional square lattice with $N=L \times L$ lattice sites, where *L* is the linear size of the lattice and the lattice sites are the nodes of the network. The lattice is divided into smaller squares of linear size $\zeta$ representing communities, e.g., cities. The number of nodes in each community is $N_c= \zeta \times \zeta$. Thus, the number of communities in our model is $n = N/N_c=L^2 / \zeta ^2$. We assume that inside a city the infection channels are dense and spread randomly between different sites in the city. Therefore, each community will be connected randomly like an Erdős–Rényi network (ER) with an average degree $k_{intra}$. In contrast, the infection channels between cities are less dense than within cities and usually connecting neighbouring cities. Thus, we assume that in addition to the intra-links linking the nodes in the same community, there are fewer inter-links which connect the nodes located in neighbouring communities. We assume that each node has inter-links distributed according to a Poisson distribution with the average degree $k_{inter} \ll k_{intra}$. Each inter-link is connected randomly to one of the nodes of the four nearest neighbouring communities occupying adjacent squares on the lattice as shown in Fig. 1. This assumption represents the fact that roads or railways usually connect neighbouring cities. For brevity of notations, we denote $K\equiv k_{intra}$ and $Q\equiv k_{inter}\zeta ^2$, where *Q* is the average number of inter-links emanating from each community to its four neighbours. To neglect the effect of the system’s edges, we used periodic boundary conditions that allow the formation of inter-links between two opposite edges of the system creating a torus structure.

**Fig. 2:**
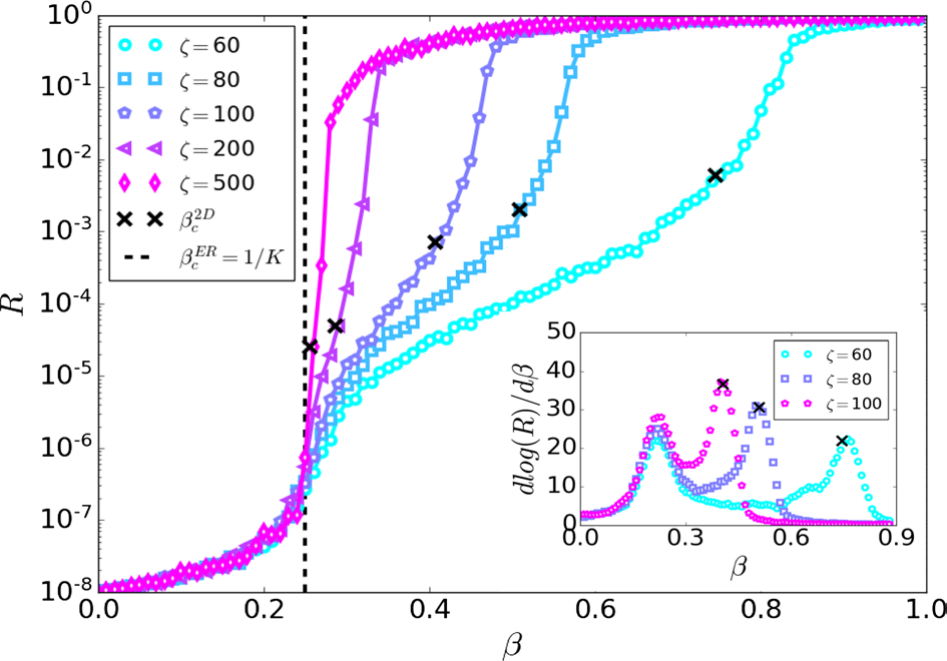
Two epidemic transitions. Simulations of the epidemic recovered cluster *R* as a function of $\beta$ for different values of $\zeta$ on a log-linear graph with $K = 4$ and $k_{inter} = 10^{-3}$. The epidemic recovered cluster is measured once no infected nodes remain. Two distinct epidemic transitions are observed. The first (lower) transition at $\beta ^{ER}_c = 1/ K$ (black dashed line) occurs when a small outbreak spread in a city but not in the entire country. The second (higher) transition at $\beta ^{2D}_c$ when a global epidemic spread in the whole country is obtained from Eq. (3) and is denoted by black $\times$. The inset shows the derivative of $\log (R)$ with respect to $\beta$ for different values of $\zeta$. Two maxima appear corresponding to the two epidemic thresholds for each $\zeta$ shown with $\times$ in the main figure. As $\zeta$ increase $\beta ^{2D}_c$ decreases and for $\zeta \rightarrow L$ the two maximums collide. Here $N = L \times L \sim 10^8$ ($L = 9960$ for $\zeta = 60$ and $L = 10^4$ for the other $\zeta$ values)

This model has two important limits. For $\zeta \rightarrow L$ the models generate an ER network while for $L \gg \zeta \rightarrow 0$ strong spatial (regular lattice) behaviour is observed. Moreover, for intermediate values of $L> \zeta > 0$ mean-field behaviour is observed in small scales (below $\zeta$) and spatial behaviour on large scales (above $\zeta$). Note that a similar but homogeneous model has been studied with similar limits (Danziger et al. 2016; Gross et al. 2017; Vaknin et al. 2017; Bonamassa et al. 2019). However, due to its homogeneous structure (and not heterogeneous as in the present modular model) it experiences very different features compared to our model with a single epidemic transition.

**Fig. 3:**
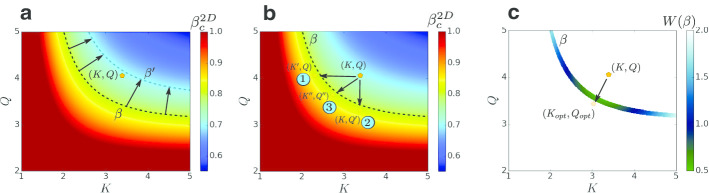
Control strategies and optimization. A given country (orange pentagon) is placed in the structural parameter space (*K*, *Q*) with epidemic threshold $\beta ^{2D}_c$ obtained from Eq. (3). For an epidemic spread with infection probability $\beta$ it is desired to position the country in such a way that $\beta < \beta ^{2D}_c(K,Q)$, such that there will be no epidemic. This can be achieved by the following strategies: **a** Social strategy. Assume that the natural epidemic infection rate is $\beta = 0.8 > \beta ^{2D}_c$ (thick black dashed line) above the epidemic threshold of the country. By using social distancing or mask-wearing the infection probability could be reduced to $\beta ^\prime = 0.7 < \beta ^{2D}_c$ (grey dashed line) and thus becomes below the epidemic threshold and the disease will not spread. **b** Quarantine strategies. By reducing the infection channels in and between the cities (reducing *K* and *Q* respectively) the country’s position in the structural parameter space can be changed and the epidemic threshold will increase such that the infection probability will be below the epidemic threshold. Three ways are suggested: (1) local quarantine strategy within cities by reducing $K \rightarrow K^{\prime }$. (2) Global quarantine strategy between cities by reducing $Q \rightarrow Q^\prime$. (3) Mixed strategy by reducing both $K \rightarrow K^{\prime \prime }$,$Q \rightarrow Q^{\prime \prime }$. **c** Strategies optimization. A weight function, $W(\beta )$, can be evaluated for optional locations for the parameters space of the country based on economical, health, and social arguments such that $\beta _c(K,Q) \rightarrow \beta ^{+}$. Optimization of the weight function will yield the optimal location for the country $(K_{opt}, Q_{opt})$. Here we used the Euclidean distance in the parameters space as a weight function $W(\beta ) = \sqrt{(K-K^{\prime \prime })^2 + (Q-Q^{\prime \prime })^2}$ and its optimization (minimizing) will yield the shortest Euclidean distance which represents minimal reduction of the inter and intra links

## Analytical and numerical results

We study the epidemic spread in our model using Monte Carlo simulations of the SIR model. In the SIR model, each node can be in one of the three states: susceptible, infected, or recovered. We start with a single infected node in a random community while all other nodes are susceptible. At each time step, every infected node attempts to infect its susceptible neighbours independently with infection probability $\beta$ and become recovered afterwards (recovery probability 1). The simulation ends when no more infected nodes remain. The impact of the epidemic outbreak for different values of $\beta$ can be measured as the fraction of the total recovered nodes in the system (the recovered cluster), *R*, once there are no more infected nodes as shown in Fig. 2. As expected, for $\zeta \rightarrow L$ the behaviour of the network approaches the behaviour of a regular ER with $\beta ^{ER}_c = 1 / K$. It can be seen that for any value of $\zeta \gg 1$ the recovered cluster has two inflection points. The first (lower) transition at $\beta ^{ER}_c$ when a local outbreak spreads within the origin city but does not propagate in the entire country. The second (higher) transition at $\beta ^{2D}_c$ when the epidemic spreads in the entire country. These two epidemic transitions are analogues to the two percolation transitions found in Gross et al. (2020c) in the same model although the analytical solution is different. While the position of the first transition does not depend on $\zeta$, the position of the second decreases with $\zeta$, and at large $\zeta$ it almost coalesces with the first one. This behaviour can be clearly seen in the derivative of $\log (R)$ as shown in the inset of Fig. 2 where the two maximum correspond to the two epidemic thresholds. As we will see, the second transition corresponds to the bond percolation threshold of the spatial network of communities which has a topology of a square lattice. This is due to the known mapping between the SIR model and bond percolation (Grassberger 1983; Sander 2002). Near this transition the epidemic spread globally in the country composed of infected cities and the size of their local outbreaks can be found analytically. Finally, near the first (lower $\beta _c$) transition corresponding to the epidemic threshold of ER network, the local outbreak disappears as well and the average recovered cluster swiftly goes to zero as $\beta$ decreases below $\beta ^{ER}_c$.

Next, we demonstrate that the second inflection point (at higher $\beta$) corresponds to the bond percolation transition on a square lattice due to the mapping from SIR (Grassberger 1983; Sander 2002). To this end we compute the position of the inflection points for different $\zeta$ analytically using the well known fact that the bond-percolation threshold for a square lattice is 1/2 (See Bunde and Havlin 1991 and “Appendix 2”). Here we will use the bond percolation threshold value to find the value of $\beta ^{2D}_c$ at which the epidemic spread in the entire country. The probability that one of *Q* inter-links emanating from a given community connects to one of its 4 neighbours is 1/4. Therefore, the number *k* of the inter-links connecting these two neighbouring communities is distributed with a binomial distribution $P_k(Q)=(1/4)^k(3/4)^{Q-k}C_Q^k$ where $C_Q^k = \frac{Q!}{k!(Q-k)!}$ is the binomial coefficient. The probability that a randomly chosen node will be part of the local outbreak in a community (city) is given by the epidemic component of ER network (See Newman 2002 and “Appendix 1”),

1 $$\begin{aligned} S = 1 - e^{-K \beta S} . \end{aligned}$$

The spread of a local outbreak in a city to one of its neighbouring cities happens through the city’s inter-links. Above $\beta ^{ER}_c$ the local epidemic spreads in the whole city and the finite non-infected clusters are of size $s\ll \zeta ^2$ and will have a very low chance to have more than one interlink for $s \cdot k_{inter}\ll 1$. Thus, assuming a very small $k_{inter}$, the probability that a local outbreak in a city will spread to one of its neighbours through a single inter-link is $S\beta$ and the probability that a local outbreak will not spread through one of the city’s inter-links is

2 $$\begin{aligned} \beta _b=\sum _k P_k(Q)(1-S \beta )^k=\left[ \frac{3}{4} + \frac{1}{4}(1 - S\beta ) \right] ^Q . \end{aligned}$$

At the lattice epidemic threshold, the probability that a local outbreak will spread to neighbouring cities, $\beta _b$ should be 1/2, the bond percolation threshold. Thus, the lattice epidemic threshold, $\beta ^{2D}_c$, where the epidemic spread in the entire country can be obtained using Eqs. (1) and (2),

3 $$\begin{aligned} \beta ^{2D}_c = \frac{4(1-2^{-1/Q})}{1 - \exp (-4K(1 - 2^{-1/Q}))} . \end{aligned}$$

Note that if the communities were distributed in a different spatial structure, the analytical approach above will still be valid but with a different value of $\beta _b$. For example, if the communities would be distributed in an hexagonal structure $\beta _b \simeq 0.6257$ (Sykes and Essam 1964).

At the spatial epidemic threshold $\beta ^{2D}_c$, the size of the local outbreak in the infected cities, $S(\beta ^{2D}_c)$, is not zero as it is usually in second order phase transitions since $\beta ^{ER}_c < \beta ^{2D}_c$ and each infected city is above criticality. The size of the local outbreak at the lattice threshold can be found analytically directly from Eqs. (1) and (2),

4 $$\begin{aligned} S(\beta ^{2D}_c) = 1 - \exp (-4K(1 - 2^{-1/Q})) . \end{aligned}$$

In the limit of $\zeta \rightarrow L$, Eq. (4) takes the form

5 $$\begin{aligned} S(\beta ^{2D}_c) \simeq \frac{4K}{Q} \ln 2, \end{aligned}$$

and $\beta ^{2D}_c = \beta ^{ER}_c = 1/K$ as expected.

**Fig. 4:**
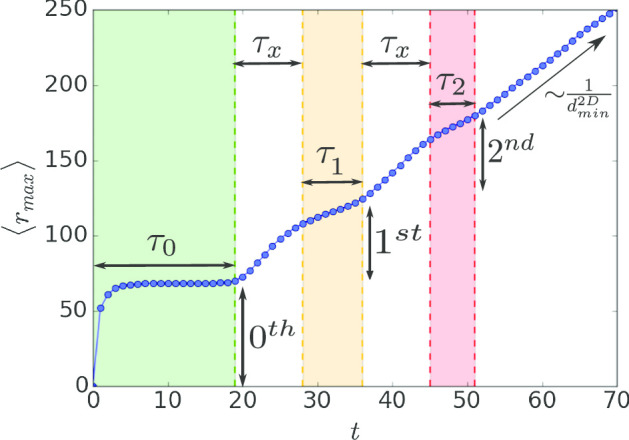
Epidemic spatial propagation. The average maximum extent of the epidemic, $\langle r_{max} \rangle$, is measured as a function of time at $\beta ^{2D}_c$. At early times the epidemic spread locally within the origin city (zeroth circle) for a period of time $\tau _0$ with a constant $\langle r_{max} \rangle \sim \zeta$. Afterwards, the epidemic spread to the first circle of cities around the origin city for a period of time $\tau _1$ and later to the second circle of cities for a period of time $\tau _2$. The transition time between the circles is denoted by $\tau _x$. As the epidemic evolves the distinction between circles decreases and identifying the distance of the disease from the origin is less clear. At later times the distinction of circles disappear completely and a clear spatial propagation is observed with $\langle r_{max} \rangle \sim t^{1/d^{2D}_{min}} = t^{1/1.13}$ (Bunde and Havlin 1991). The reason for the disappearance of the distinction between circles at later times is because the epidemic may spread faster in a given area and slower in another leading to inconclusive distinction between circles. Here we used $\zeta = 100$, $K = 4$, $k_{inter} = 10^{-3}$, $L = 1000$ and $\beta ^{2D}_c = 0.407$

**Fig. 5:**
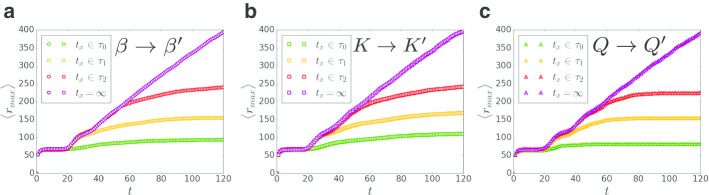
Intervention timing. Here we show the effect of different control strategies performed at intervention timing $t_x$ at different circles’ timescales corresponding to Fig. 4 and compare them to the scenario of no intervention corresponding to the case of $t_x = \infty$. The epidemic start spreading with the parameters $\zeta = 100$, $K = 4$, $k_{inter} = 10^{-3}$, $Q = 10$, $L = 1000$ and $\beta = \beta _c = 0.407$. **a** Social strategy. $\beta \rightarrow \beta ^\prime = 0.3$. **b** Quarantine strategy within cities. $K \rightarrow K^\prime = 3$. **c** Quarantine strategy between cities. $Q \rightarrow Q^\prime = 1$ by reducing $k_{inter} \rightarrow k_{inter}^\prime = 10^{-4}$ and keeping $\zeta$ fixed. The epidemic extent at $t_x$ is $\langle r_{max} \rangle _x$ and when the epidemic stops to spread it is $\langle r_{max} \rangle _f$. In all cases the intervention successfully stop the disease spatial propagation with $\langle r_{max} \rangle _f \approx \langle r_{max} \rangle _x$

## Control strategies and optimization

When an epidemic spreads in a country it is essential to develop control strategies to tame the disease. For this purpose it is very insightful to study the phase diagram in the structural parameters space (*K*, *Q*) (Fig. 3). A given country is placed in the structural parameter space according to its inter-degree *K* and city intra-degree *Q* for which its epidemic threshold can be calculated from Eq. (3). For the case of an epidemic with infection probability $\beta > \beta ^{2D}_c(K,Q)$ the epidemic will spread in the country and an appropriate control strategy should be considered. This scenario can be visually observed in the parameter space when the system is placed above the line $\beta = \beta ^{2D}_c(K,Q)$ (Fig. 3a black dashed line). Since $\zeta$ is related to the spatial structure of the cities and rarely changes during the timescale of the epidemic, one should try effecting the other parameters in order to control the disease. The main goal is to achieve a state such that $\beta < \beta ^{2D}_c(K,Q)$ and the epidemic will not spread. In such a case the system will be placed below the line $\beta = \beta ^{2D}_c(K,Q)$ (Fig. 3a grey dashed line). This can be achieved through the following strategies:

*Social strategy.* Since the epidemic propagates through human interactions, the basic approach could be based on reducing the infection probability [which has been applied in another context (Braha and Bar-Yam 2007)] $\beta \rightarrow \beta ^\prime$ in such a way that $\beta ^{\prime } < \beta ^{2D}_c(K,Q)$ as shown in Fig. 3a. This can be achieved by social distancing, mask-wearing, etc to reduce the probability of an infected person to infect others. This approach and its effectiveness vary between countries and populations due to many factors such as population dynamics.

*Quarantine strategy.* In the case that social strategies are not effective enough and after applied still $\beta ^{\prime } > \beta ^{2D}_c(K,Q)$, one can apply quarantine strategies by reducing the infection channels in and between cities. The first approach reduces the degree within cities $K \rightarrow K^\prime$ such that $\beta < \beta ^{2D}_c(K^\prime ,Q)$ as shown in the first option in Fig. 3b. $K^\prime$ should be reduced below the critical value $K_c$ obtained from $\beta = \beta ^{2D}_c(K_c,Q)$ which can be analytically found from Eq. (3),

6 $$\begin{aligned} K_c = -\frac{1}{4(1-2^{-1/Q})} \log \left[ 1 - \frac{4(1-2^{-1/Q})}{\beta }\right] . \end{aligned}$$

The second approach reduces the degree between cities $Q \rightarrow Q^\prime$ (through $k_{inter}$ since $\zeta$ is usually fixed) such that $\beta < \beta ^{2D}_c(K,Q^\prime )$ as shown in the second option in Fig. 3b. The value of $Q^\prime$ should be reduced below the critical value $Q_c$ which can be graphically evaluated from Eq. (3).

The third approach involve combining the above two options by reducing both the degree in and between cities $(K,Q) \rightarrow (K^{\prime \prime },Q^{\prime \prime })$ such that $\beta < \beta ^{2D}_c(K^{\prime \prime },Q^{\prime \prime })$ as shown in the third option in Fig. 3b.

When considering which approach to adopt, an optimization method can be developed. To this end, a weight function $W(\beta )$ can be evaluated for optional locations for the country in the parameters space based on economical, health, and social arguments. $W(\beta )$ is evaluated on the $\beta _c(K,Q) \rightarrow \beta ^{+}$ line as shown in Fig. 3c. Optimization of the weight function will yield the optimal location for the country $(K_{opt}, Q_{opt})$ in the parameter space. In Fig. 3c we used the Euclidean distance in the parameters space as a weight function $W(\beta ) = \sqrt{(K-K^{\prime \prime })^2 + (Q-Q^{\prime \prime })^2}$ and its optimization (minimizing) will yield the shortest Euclidean distance which represents the minimal reduction of the inter and intra links, i.e., minimal restrictions. However, in a real scenario much more complex function is required. This function should take into account the economic cost of reducing the degree in and between the cities, social cost of quarantine, and many other collateral damage factors.

## Consequences of late intervention and early quarantine removal

While well-performed control strategies (Fig. 3) will result in epidemic extinction, the timing of the intervention plays a significant role. In many cases, early action can control the disease rapidly with a low amount of infections while late reply may not be efficient since the epidemic may already spread globally. To understand the effect of intervention timing we study the spatial propagation of the epidemic as a function of time. In Fig. 4 we show the average maximal extent of the epidemic from the origin, $\langle r_{max} \rangle$, as a function of time at $\beta ^{2D}_c$. The value of $\langle r_{max} \rangle (t)$ is measured as the average of the maximum distance from the disease origin of the newly recovered nodes until time *t*, and describes the spatial propagation of the disease. Assuming a small $k_{inter}$, at early times the spatial structure of cities around the origin city can be observed in the spatial propagation of the diseases. In the beginning, the disease spread locally within the origin city (zeroth circle) with $\langle r_{max} \rangle \sim \zeta$. The timescale of this stage is $\tau _0$ which generally depends on $\zeta$, *K*, and $k_{inter}$. Afterwards, the epidemic spread in the first circle of cities around the origin city with timescale $\tau _1$ and later in the second circle with timescale $\tau _2$. $\tau _x$ is the timescale of crossing between circles. Interestingly, the timescale of the disease spread in each circle gets shorter as the disease spread further and eventually completely disappear. At this point, a clear 2D spatial propagation (of fractal type-since the system is at criticality) is observed with $\langle r_{max} \rangle \sim t^{1/d^{2D}_{min}} = t^{1/1.13}$ (Bunde and Havlin 1991) as shown in Fig. 4. The reason for the disappearance of the distinction between circles at later times is because the epidemic may spread faster in a given area and slower in another leading to inconclusive distinction between circles.

**Fig. 6:**
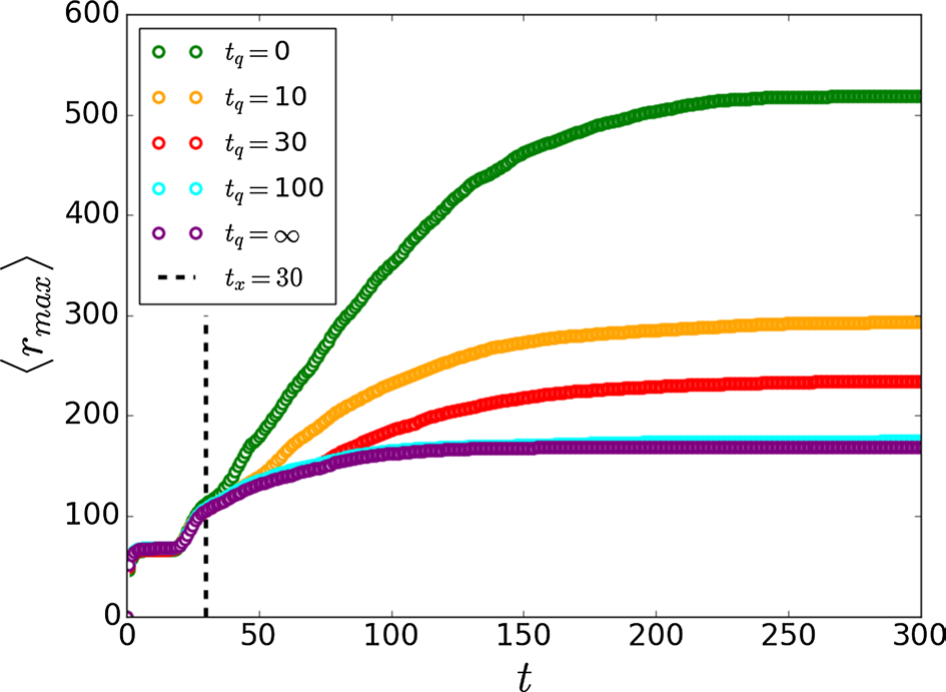
Temporal quarantine strategy. Here we show the effect of quarantine strategy within cities (Fig. 5b) for different quarantine time windows $t_q$. At time $t_x = 30$ we temporally reduce $K \rightarrow K^{\prime }$ within cities (black dashed line) and at time $t_x + t_q$ remove the quarantine $K^{\prime } \rightarrow K$. The quarantine window highly affects the final extent of the epidemic, $\langle r_{max} \rangle _f$, and while early removal of the quarantine will result with the epidemic still propagating in the system, a finite quarantine window can completely stop the propagation of the disease with $\langle r_{max} \rangle _f \approx \langle r_{max} \rangle _x$. Here we used $\zeta = 100$, $K = 4$, $K^{\prime } = 3$, $k_{inter} = 10^{-3}$, $L = 1000$ and $\beta = \beta ^{2D}_c = 0.407$

This phenomenon of spatial identification of the disease in different circles at early times and its disappearance at later times has significant consequences for intervention timing. At early times when the epidemic can be identified in a given circle, quarantine strategies (Fig. 3b) can be applied locally around the circle and control the disease without affecting the whole country [as could have been done around Hubei province in China in the case of COVID-19 (Gross et al. 2020a)]. In contrast, in later times the identification of the disease location becomes inconclusive and intervention should be applied on much larger scales to control the disease. Moreover, while in the close circles the spatial identification is valid as shown in Fig. 4 for the first and the second circles, the time window for action gets shorter and shorter ($\tau _0> \tau _1 > \tau _2$) and require fast decisions. In Fig. 5 we tested different control strategies discussed in Fig. 3 applied in different timing, $t_x$. In order to test if the intervention was successful, it is of interest to compare $\langle r_{max} \rangle _x$, the epidemic extent at $t_x$, and the epidemic extent once the epidemic stop spreading $\langle r_{max} \rangle _f$. As shown in Fig. 5 the intervention was successful in controlling the disease with $\langle r_{max} \rangle _f \approx \langle r_{max} \rangle _x$. Nonetheless, while the control strategies at early times successfully stop the disease propagation, the extent of action required to control the disease increases as the intervention is performed later resulting with a larger amount of infections as the epidemic spreads further. These results highlight the importance and impact of early actions.

To complete the picture we also analyze the effect of temporal quarantine strategy by temporally reducing the degree within cities (Fig. 5b, $K \rightarrow K^{\prime }$) at time $t_x$ and increasing it back after quarantine window of $t_q$ ($K^{\prime } \rightarrow K$). Figure 6 show the effect of temporal quarantine within cities for different quarantine windows. As can be seen, the quarantine window highly affect the final extent of the epidemic, $\langle r_{max} \rangle _f$, and while early removal of the quarantine will result with the epidemic still propagating in the system, a finite quarantine window can completely stop the propagation of the disease with $\langle r_{max} \rangle _f \approx \langle r_{max} \rangle _x$. Note that longer quarantine windows will be required for lower recovery probability. These results show that while early actions are essential, early removal of them will not result with the control of the disease and therefore quarantine removal should be perform with maximum caution.

## Summary and discussion

In this work, we applied the SIR model to study the epidemic spreading on a spatial modular network model which can represent cities in a country. We find that two epidemic thresholds exist, the first representing a local outbreak within a city, and the second when the epidemic spreads globally in the entire country. We find analytically both epidemic thresholds and based on them we developed control strategies and a method to optimize them. The first strategy uses social measures to reduce the infection probability, and the second uses quarantine measures by reducing the infection channels within and between the cities. We also study the effect of intervention timing and show that early actions are essential to prevent the global spread of the disease.

Our model provides an analytical solution for epidemic spreading in spatial system, however, in order to achieve that, some simplifications were applied and should be improved in future work. The first is the assumption that all cities have the same size $\zeta ^2$ which in fact should follow a certain realistic distribution. Nonetheless, we expect qualitatively similar results as we showed here. The second is the absence of long-range connections. While in some cases such as a epidemic outbreak when quarantine are applied this assumption is valid since only short range connection is allowed, future work should also include realistic long range links into consideration which could be related to the Watts-Strogatz model (Watts and Strogatz 1998). Despite these simplifications, our model, control and optimization strategies will still be applicable when including more realistic features.

In addition, another direction for future work is a spectral analysis of the network model which has been shown to explain many phenomena in percolation (Bollobás et al. 2010) and may provide a new perspective to the phenomena we observed here. Moreover, it may assist in the effort of spectral analysis study of clustered networks which is not yet fully understood today (Zhang 2017).

## Figures and Tables

**Fig. 7:**
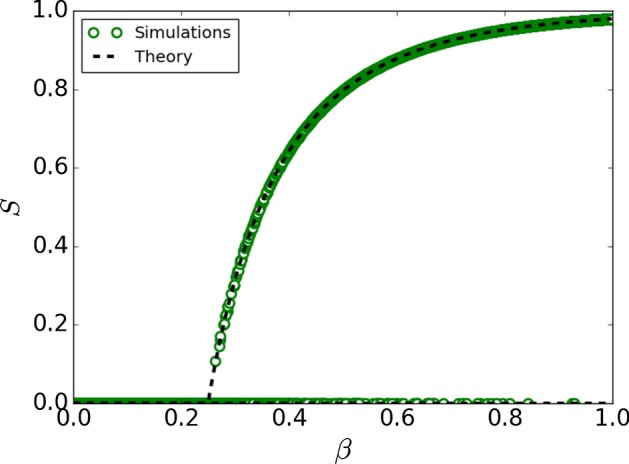
Epidemic spread in ER networks. Theory is obtained from Eq. (12) and simulation performed with $N = 10^6$ and $K = 4$. The epidemic threshold is $\beta ^{ER}_c = 1/K = 1/4$

**Fig. 8:**
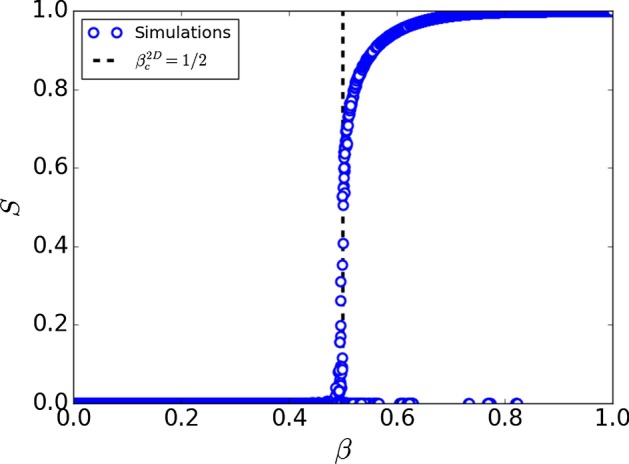
Epidemic spread in 2D square lattice. The simulations were performed for $N = L \times L$ with $L = 1000$. The epidemic threshold is $\beta ^{2D}_c = 1/2$

## Data Availability

Not applicable.

## Appendix 1 Epidemic spread in ER networks

To develop an analytical solution for epidemic spread in ER networks we will follow the formalism developed by Newman (2002). We will use the generating functions:

7 $$\begin{aligned} G_0(u)&= \sum _k p_k u^k, \end{aligned}$$

8 $$\begin{aligned} G_1(u)&= \frac{1}{K}G^\prime _0(u) . \end{aligned}$$

for the degree distribution and the outgoing edges distribution respectively. In order to find the size of the epidemic outbreak we need the generating functions for the distribution of the *occupied edges*. Thus, following Newman (2002) the generating function for the occupied edges for epidemic with infection probability $\beta$ is:

9 $$\begin{aligned} G_0(u;\beta ) = G_0(1 + (u-1)\beta ). \end{aligned}$$

and

10 $$\begin{aligned} G_1(u;\beta ) = G_1(1 + (u-1)\beta ). \end{aligned}$$

For the case of ER networks $p_k = \frac{K^ke^{-K}}{k!}$, thus:

11 $$\begin{aligned} G_0(u;\beta ) = G_1(u;\beta ) = e^{-K\beta (1-u)} \end{aligned}$$

where *K* is the average degree. The size of the epidemic outbreak is $S(\beta ) = 1- G_0(u;\beta )$ and $u(\beta ) = G_1(u;\beta )$. Thus, $S = 1-u$ and a self consistent equation can be written:

12 $$\begin{aligned} S = 1 - e^{-K \beta S} \end{aligned}$$

with the epidemic threshold $\beta ^{ER}_c = 1/K$. Theory and simulation show excellent agreement as shown in Fig. 7.

## Appendix 2 Epidemic spread in 2D square lattice

The mapping between bond percolation and the SIR model yield the same epidemic threshold for 2D square lattice $\beta ^{2D}_c = 1/2$ (Grassberger 1983; Sander 2002). Simulations of the SIR model on a 2D square lattice are shown in Fig. 8 with the epidemic threshold $\beta ^{2D}_c = 1/2$ as expected.

## Abbreviations

SIR: Susceptible-infected-recovered
ER: Erdős–Rényi network
